# Gastrointestinal Nematode-Derived Antigens Alter Colorectal Cancer Cell Proliferation and Migration through Regulation of Cell Cycle and Epithelial-Mesenchymal Transition Proteins

**DOI:** 10.3390/ijms21217845

**Published:** 2020-10-22

**Authors:** Brittany-Amber Jacobs, Sharon Prince, Katherine Ann Smith

**Affiliations:** 1 Institute of Infectious Disease and Molecular Medicine, University of Cape Town, Cape Town 7925, South Africa; brittany-amber.jacobs@ludwig.ox.ac.uk; 2 Department of Human Biology, University of Cape Town, Cape Town 7925, South Africa; sharon.prince@uct.ac.za; 3 School of Medicine, Cardiff University, Cardiff CF14 3XN, UK

**Keywords:** colorectal cancer, gastrointestinal nematode, proliferation, migration

## Abstract

As the global incidences of colorectal cancer rises, there is a growing importance in understanding the interaction between external factors, such as common infections, on the initiation and progression of this disease. While certain helminth infections have been shown to alter the severity and risk of developing colitis-associated colorectal cancer, whether these parasites can directly affect colorectal cancer progression is unknown. Here, we made use of murine and human colorectal cancer cell lines to demonstrate that exposure to antigens derived from the gastrointestinal nematode *Heligmosomoides polygyrus* significantly reduced colorectal cancer cell proliferation in vitro. Using a range of approaches, we demonstrate that antigen-dependent reductions in cancer cell proliferation and viability are associated with increased expression of the critical cell cycle regulators p53 and p21. Interestingly, *H. polygyrus*-derived antigens significantly increased murine colorectal cancer cell migration, which was associated with an increased expression of the adherens junction protein β-catenin, whereas the opposite was true for human colorectal cancer cells. Together, these findings demonstrate that antigens derived from a gastrointestinal nematode can significantly alter colorectal cancer cell behavior. Further in-depth analysis may reveal novel candidates for targeting and treating late-stage cancer.

## 1. Introduction

Globally, colorectal cancer (CRC) is the third most commonly diagnosed malignancy and the second leading cause of cancer deaths [1]. In 2018 alone, CRC represented 1.8 million new worldwide cancer cases and 900,000 deaths. It has been suggested that the lifestyle that is common in more developed countries, including a diet low in fiber, fruits, and vegetables, obesity, lack of physical activity, excessive alcohol consumption, and a lack of sleep, increases the risk of developing CRC [2,3]. Furthermore, chronic inflammation associated with inflammatory bowel disease (IBD) has been linked to an increased risk of high-grade dysplasia and cancer, known as colitis-associated colorectal cancer (CAC) [4,5].

Notably, many soil-transmitted helminths (STH) establish life-long infections associated with chronic inflammation within the gastrointestinal tract. During persistent infection, parasitic helminths release excretory/secretory products containing components that can modify systemic and local inflammatory responses [6]. Both helminth infections and their associated antigens have been shown to alter the development and progression of CAC [7,8,9,10,11]. Despite this, it is unclear whether these parasites can directly impact CRC progression. Helminths infect over 1 billion individuals in low- and middle-income countries and result in the loss of 20 million disability-adjusted life years (the amount of years lost due to illnesses, disability, or premature death) [12,13]. The gastrointestinal tract of an individual living in these regions is likely to be parasitized by one, if not all three, of the leading STH: roundworms, hookworms, and whipworms [14]. Globally, 807 million people are infected with roundworms, 700 million are infected with hookworms and 604 million are infected with whipworms [14].

Certain helminth infections have been shown to confer an advantage to the host, by reducing autoimmune and allergic symptoms in infected individuals [15,16,17,18]. However, others have been reported to diminish vaccine efficacy and impair host immune responses to co-infection [19,20,21]. Moreover, although only partially understood, helminth infections can also influence the risk of cancer development. For example, *Opisthorchis viverrini* and *Clonorchis sinensis* are classified as group one biological carcinogens and are conclusive causes of cholangiocarcinoma (CCA), also known as bile duct cancer [22,23]. Opisthorchis-induced CCA is thought to occur due to damage to the bile duct epithelium, cell proliferation, and inhibition of DNA repair and apoptosis, which are a consequence of mechanical damage caused by feeding parasites, immunopathology and the effects of fluke secreted proteins [24].

Chronic infection with Schistosoma has been associated with the development of bladder cancer, increased chromosomal aberrations, and increased DNA copy number [25,26]. Increased CRC was also observed in patients with a chronic Schistosoma or gastrointestinal parasitic infection [27,28]. Accumulating evidence in pre-clinical murine models of CRC suggests that differing STH infections can have diverse effects on CRC initiation by exacerbating neoplastic change and tumor formation or inducing intestinal epithelial cell remodeling [29,30]. Although increased CRC following Schistosoma infection has been associated with the deposition of Schistosomal ova, which has been shown to contain oncogenic antigens, it is not currently known how STH antigens influence CRC progression [31,32].

Here, we made use of antigen derived from *Schistosoma mansoni* and *Heligmosomoides polygyrus* to determine how STH antigens impact CRC cell proliferation and migration. We reveal that antigen derived from *H. polygyrus* significantly decreased murine and human CRC cell proliferation, which was associated with increased expression of p53 and p21. Furthermore, exposure to these antigens reduced both murine and human CRC cell mitochondrial activity. *H. polygyrus*-derived antigens increased murine CRC cell migration, which was associated with increased expression of the adherens junction protein β-catenin. Interestingly, the opposite was true for human colorectal cancer cells. These data demonstrate the usefulness of in vitro assays in assessing how differing parasite antigens impact on CRC cell behavior and identify pathways that are altered in CRC cells following exposure to helminth antigens.

## 2. Results

### 2.1. H. polygyrus-Derived Antigens Limit CRC Cell Proliferation and DNA Synthesis

**Figure 1:**
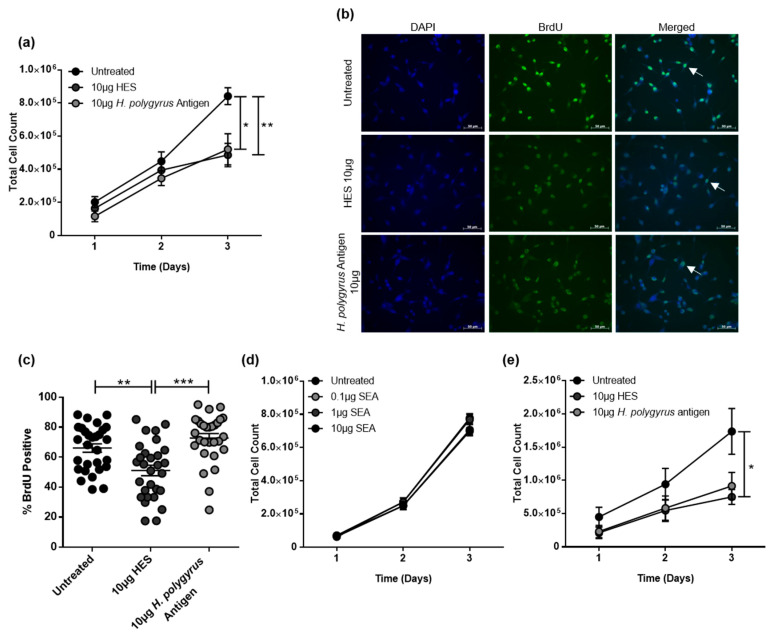
*H. polygyrus*-derived antigens limit colorectal cancer (CRC) cell proliferation. Live cell counts over a 3-day period of (**a**) CT26.WT and (e) HCT116 cells, following exposure to 10 µg *H. polygyrus* excretory/secretory products (HES) or 10 µg *H. polygyrus* adult antigen; (**b**) BrdU incorporation (green) was determined in live cells (blue DAPI nuclear stain) 8 h after addition of 10 µg HES or 10 µg *H. polygyrus* antigen to CT26.WT cells; (**c**) quantification of the BrdU positive stain within live cells (white arrow in b); (**d**) live cell counts over a 3-day period, following exposure of CT26.WT cells to 0.1 µg, 1 µg, and 10 µg *Schistosoma mansoni* egg antigen (SEA). The data is representative of four (**a**), three (**d,e**) and two (**b,c**) independent experiments. Significance was analysed using GraphPad Prism 6.01, and a one-way ANOVA (**a**,**b**,**d**) or Kruskal–Wallis test (**e**) was performed. * *p <* 0.05, ** p < 0.01, and *** p < 0.001. Error bars represent the standard error of the mean. The scale bar is 50 µm.

Presently, the effect of exposure to STH antigens on CRC cell proliferation is not known. We therefore exposed murine CT26.WT CRC cells to 10 μg antigen from *H. polygyrus* adults and the excretory/secretory products (HES) and quantified the impact on cell viability using trypan blue exclusion. Our results reveal that, compared to untreated cells, there was a significantly decreased number of viable treated CT26.WT cells (Figure 1a). To confirm the results seen in Figure 1a, a BrdU incorporation assay was performed, which quantifies DNA synthesis in replicating (cycling) cells. The results demonstrated that, while HES treatment significantly reduced DNA synthesis in CT26.WT cells, there was no significant effect observed for *H. polygyrus* antigen treatment (Figure 1b,c). Surprisingly, increasing doses of the egg antigen derived from *Schistosoma mansoni* ova (SEA) had no effect on CT26.WT cell proliferation (Figure 1d). We then confirmed that 10 μg of HES significantly decreased the number of viable human CRC cells and limited human CRC cell proliferation using the cell line HCT116 (Figure 1e). With this cell line, no significant effect on proliferation was observed for *H. polygyrus* antigen treatment (Figure 1e).

### 2.2. H. polygyrus Excretory/Secretory Products Significantly Decrease CRC Cell Viability in a Species-Specific Manner

**Figure 2:**
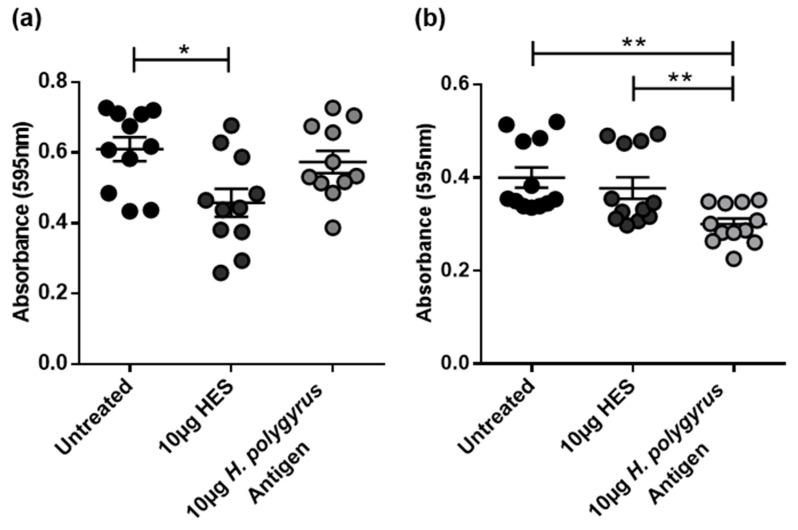
*H. polygyrus*-derived antigens decrease colorectal cancer (CRC) cell viability. The cell viability of (**a**) CT26.WT and (**b**) HCT116 cells exposed to 10 µg HES or 10 µg *H. polygyr*us antigen for 48 h was assessed by 3-(4,5-dimethylthiazol-2-yl)-2,5-diphenyltetrazolium bromide (MTT) assay. MTT was added to each well for 4 h, followed by the addition of the solubilizer, and 24 h later, the absorbance of each well was read at a wavelength of 595 nm. The data are representative of three independent experiments. Significance was analysed using GraphPad Prism 6.01 and a one-way ANOVA was performed, where * *p* < 0.05 and ** *p* < 0.01. Error bars represent the standard error of the mean.

Cell proliferation is defined as a balance between cell division and cellular differentiation or cell death. In order to further determine the factors contributing to the significant reduction in cell proliferation and DNA synthesis seen in Figure 1a–d, an 3-(4,5-dimethylthiazol-2-yl)-2,5-diphenyltetrazolium bromide (MTT) assay was performed to quantify the mitochondrial activity as a measure of cell viability. Consistent with the BrdU results, incubation with HES significantly reduced the cell viability in murine CT26.WT cells. However, no significant effect was observed for *H. polygyrus* antigen treatment (Figure 2a), nor for treatment of human HCT116 CRC cells with HES (Figure 2b).

### 2.3. H. polygyrus-Derived Antigens Increase the Expression of the Cell-Cycle Arrest Proteins p21 and p53 in CRC Cells

**Figure 3:**
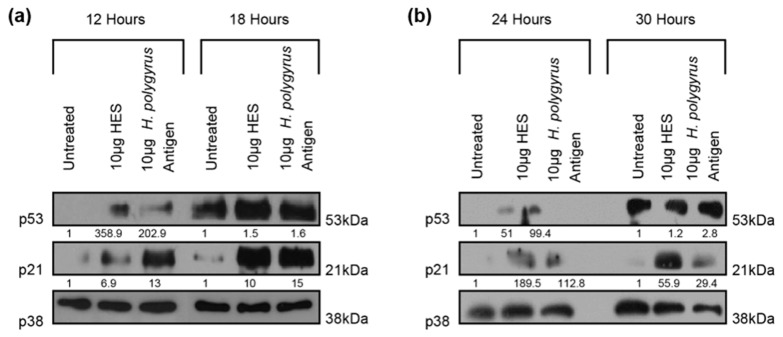
*H. polygyrus*-derived antigens increase the expression of colorectal cancer (CRC) cell-cycle arrest proteins p53 and p21. Western blot analyses of p53, p21, and p38 (loading control) were performed following exposure of (**a**) CT26.WT cells for 12- and 18-h and (**b**) HCT116 cells for 24- and 30-h to 10 µg HES or 10 µg *H. polygyrus* antigen. Densitometry readings were obtained using ImageJ in order to determine the changes in protein expression of p53 and p21 normalized to p38 and expressed as a fold-change relative to control wells. The data is representative of two independent experiments.

In response to cellular stress, the tumor suppressor p53 is activated and, in order to prevent the proliferation of damaged cells, it transactivates its target genes that control cell-cycle arrest, senescence, and/or apoptosis [33]. Targets of p53 include p21, a cyclin-dependent kinase inhibitor, which induces cell-cycle arrest [34]. Given the impact of *H. polygyrus*-derived antigens on the proliferation of human and murine cells lines (Figure 1a or Figure 1e), we next examined whether this activity was related to changes in p53 and p21. Results from two independent experiments demonstrate that both *H. polygyrus* antigen and HES elevated the expression of p53 and p21 in murine CT26.WT (Figure 3a) and in human HCT116 (Figure 3b) cells (Supplementary Materials, Figure S1). In CT26.WT cells, HES and *H. polygyrus* antigen increased the expression of p53 at 12 h (mean 183.65 fold, SD 247.8, and mean 105.85 fold, SD 137.2, respectively) and at 18 h (mean 1.5 fold, SD 0, and mean 2.45 fold, SD 1.2, respectively) (Figure 3a top panel; Supplementary Materials: Figure S1a top panel). The expression of p21 was also increased following exposure to HES and *H. polygyrus* antigen at 12 h (mean 7.15 fold, SD 0.4, and mean 9.35 fold, SD 5.2, respectively) and at 18 h (mean 5.55 fold, SD 6.3, and mean 8.2 fold, SD 9.6, respectively) (Figure 3a middle panel; Supplementary Materials: Figure S1a middle panel). In HCT116 cells, HES and *H. polygyrus* antigen increased the expression of p53 at 24 h (mean 26.8 fold, SD 34.2, and mean 95.3 fold, SD 5.8, respectively) and at 30 h (mean 1.8 fold, SD 0.9, and mean 2.4 fold, SD 0.6, respectively) (Figure 3b top panel; Supplementary Materials: Figure S1b top panel). The expression of p21 was increased by HES and *H. polygyrus* antigen at 24 h (mean 95.7 fold, SD 132.7, and mean 57.35 fold, SD 78.4, respectively) and at 30 h (mean 28.65 fold, SD 38.5, and mean 16.05 fold, SD 18.9, respectively (Figure 3b middle panel; Supplementary Materials: Figure S1b third panel).

### 2.4. H. polygyrus-Derived Antigens Increase Murine CRC Cell Migration and β-Catenin Expression

**Figure 4:**
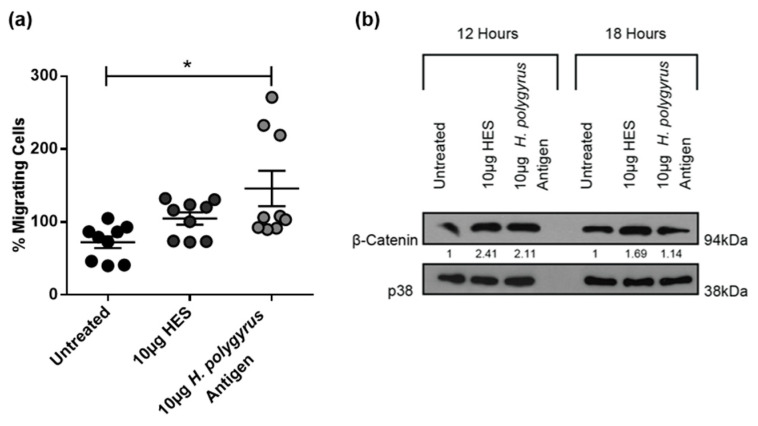
*H. polygyrus*-derived antigens increase murine colorectal cancer (CRC) cell migration and β-catenin expression. (**a**) Transwell migration assay of untreated control and CT26.WT cells exposed to 10 µg HES or 10 µg *H. polygyrus* antigen for 24 h; (**b**) Western blot analysis of β-catenin and p38 (loading control) was performed 12- or 18-h following exposure of CT26.WT cells to 10 µg HES, 10 µg *H. polygyrus* antigen, or an untreated control. Densitometry readings were obtained using ImageJ in order to determine the changes in protein expression of β-catenin normalised to p38 and expressed as a fold-change relative to control wells. The data is representative of three (**a**) and one (**b**) independent experiments. Significance was calculated using GraphPad Prism 6.01 and a one-way ANOVA was performed, where * p < 0.05. Error bars represent the standard error of the mean.

Cell migration is a fundamental step in CRC progression and is associated with poor outcome. We therefore next assessed whether the *H. polygyrus*-derived antigens would alter CRC cell migration using a transwell migration assay. We demonstrate that *H. polygyrus* antigen significantly increased CT26.WT cell migration, whereas there was a trend toward increased migration following exposure to HES (Figure 4a). The aberrant expression and accumulation of β-catenin is a signature of CRC cell patients [35]. This dual function protein, involved in cell-cell adhesion and transcription, is thought to promote invasion, metastasis, and dormancy in human colorectal tumors [36]. Results from one western blot experiment suggest that increased CT26.WT cell migration, following treatment with *H. polygyrus*-derived antigens, was associated with increased expression of β-catenin (Figure 4b). The expression of β-catenin was increased by HES and *H. polygyrus* antigen at 12 h by 2.41 and 2.11-fold, respectively, and at 18 h by 1.69 and 1.14-fold, respectively.

### 2.5. H. polygyrus-Derived Antigens Influence CRC Cell Migration and β-Catenin Expression in a Species-Specific Manner

**Figure 5:**
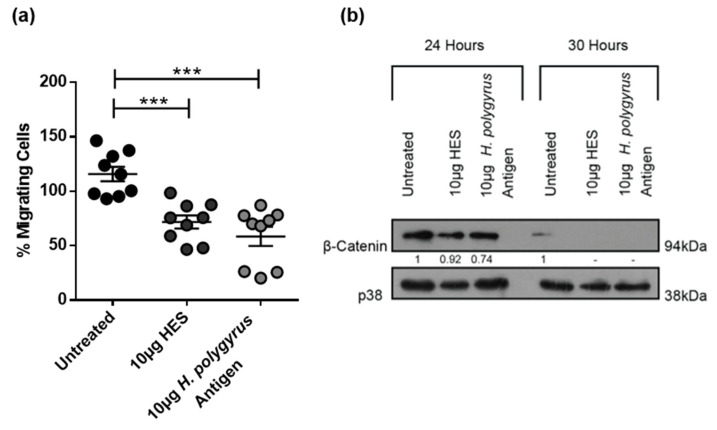
*H. polygyrus*-derived antigens decrease human colorectal cancer (CRC) cell migration and β-catenin expression. (**a**) Transwell migration assay of HCT116 cells determined at 24 h, following exposure to 10 µg HES, 10 µg *H. polygyrus* antigen, or an untreated control; (**b**) Western blot analysis of β-catenin and p38 (loading control) was performed 24- or 30-h following exposure of HCT116 cells to 10 µg HES, 10 µg *H. polygyrus* antigen, or an untreated control. Densitometry readings were obtained using ImageJ in order to determine the changes in protein expression of β-catenin normalized to p38 and expressed as a fold-change relative to control wells. The data is representative of three (**a**) and one (**b**) independent experiments. Significance was analyzed using GraphPad Prism 6.01 and a one-way ANOVA was performed, where *** *p <* 0.001. Error bars represent the standard error of the mean.

Interestingly, unlike the data obtained for the murine CT26.WT cells, transwell migration assay results showed that HES and *H. polygyrus* antigen significantly reduced the migration of human colorectal cancer cells (Figure 5a). Consistent with their inhibitory effect on the migration of HCT116 cells, results from one Western blot experiment suggest that *H. polygyrus*-derived antigens decreased β-catenin levels expressed by these cells (Figure 5b). The expression of β-catenin was decreased by HES and *H. polygyrus* antigen at 24 h by 0.92 and 0.74-fold, respectively, and to below detectable levels, at 30 h.

## 3. Discussion

Although certain helminths have been shown to modulate the host immune response by promoting immune-regulatory mechanisms, others have been classified as biological carcinogens and are irrefutable causes of cancer [37,38]. Currently, evidence for how helminth infections and their associated antigens impact on the risk of cancer development is still emerging. In this study, we aimed to determine the effect of soil-transmitted helminths (STH) antigen on colorectal cancer (CRC) cell behavior.

Accumulating reports suggest that helminth infections can exacerbate the initiation of CRC; however, whether infection or associated helminth antigens impact on CRC development is still unclear [9,27,28,29,32,39]. Our results demonstrate that *H. polygyrus* antigen and *H. polygyrus* excretory/secretory products (HES) significantly reduced the in vitro proliferation of murine (CT26.WT) CRC cells, while HES alone significantly reduced the in vitro proliferation of human (HCT116) CRC cells. This result supports previous studies with a *Trichinella spiralis*-derived antigen, which inhibited the proliferation of human myeloid leukemia and hepatoma cells in vitro, as well as the proliferation of intestinal cells in vivo [40,41,42]. However, they are in opposition to one study with the ES products from the carcinogenic *O. viverrini*, which increased the proliferation of fibroblasts in vitro [41].

The mechanism for how *H. polygyrus*-derived antigens reduced CRC proliferation is unclear; however, BrdU incorporation and MTT assays demonstrated that HES significantly reduced DNA synthesis and cell viability, respectively, in CT26.WT cells. These data suggest that the excretory/secretory antigens are able to shift CRC cells from proliferation to a more quiescent phenotype. This is in accordance with the findings using *Trichinella spiralis*-derived antigen, which arrested cancer cell lines at G1 or S phase of the cell cycle [41]. Interestingly, we found that *H. polygyrus* antigen did not have a profound impact on CT26.WT DNA synthesis or cell viability of CRC cells, despite a significant impact on cancer cell proliferation. *H. polygyrus*-derived antigens contain different mixtures of proteins and glycoproteins, as well as small RNAs, all of which can modulate host cell behavior [6,43,44,45]. Further analysis of the individual components of *H. polygyrus* adult antigen and HES would need to be undertaken in order to determine whether the presence of varying components may account for the contrasting effects of these antigens on differing aspects of cell proliferation.

Western blot analyses showed that a significant reduction in proliferation of human and murine CRC cells, following exposure to *H. polygyrus*-derived antigens, was associated with an upregulation of p53 and p21 expression; molecules that are associated with increased cell cycle arrest and apoptosis. Chronic infection with *Taenia crassiceps* and *Trichinella spiralis* differentially regulated local expression of p53 in vivo, whereas exposure of hepatic stellate cells to *Schistosoma japonicum* soluble egg antigen induced apoptosis following upregulation of p53 [46,47,48]. In the latter publication, *Schistosoma japonicum* soluble egg antigen induction of apoptosis was associated with inhibition of Akt signaling and subsequent upregulation of p53-dependent DR5 expression. HES has previously been described as anti-apoptotic because it significantly reduced annexin V and propidium iodine staining in proliferating CD4^+^ T cells [49]. Given our findings, decreased proliferation of CRC cells, following exposure to HES, is more likely to represent an increase in cell cycle arrest (quiescence) than increased apoptosis of these cells.

Surprisingly, *H. polygyrus* antigen and HES caused an increase in CT26.WT cell migration, which was supported by changes in β-catenin expression. β-catenin (NCBI Gene ID: 1499) forms part of the adherens junctions between cells and is negatively regulated by adenomatous polyposis coli (APC) [50]. Significantly, 85% of CRC cancers are associated with mutations in the APC gene, resulting in a loss of its tumor suppressor function [50,51]. As a result, β-catenin expression becomes upregulated and acts as an oncoprotein. This activation of the Wnt signaling pathway, of which APC and β-catenin are a part, has been implicated in initiating CRC development and is associated with poor prognosis [7,50,52]. It is thus anticipated that the elevated level of expression of β-catenin in treated CT26.WT cells coincides with a significant increase in cell migration. Importantly, live infection and in vivo and in vitro treatments with helminth-derived antigens, have previously been shown to alter the expression of epithelial-mesenchymal transition (EMT) markers, including β-catenin [7,10,53,54]. In contrast to results observed for the murine CT26.WT cell line, *H. polygyrus*-derived antigens caused a significant decrease in human HCT116 cell migration, which corresponded with a decrease in β-catenin expression.

While it is possible that multiple components of *H. polygyrus*-derived antigens may alter cancer cell behavior in a number of ways, proteomics analysis of these antigens, combined with sequencing of the *H. polygyrus* genome, has led to the identification of several immunomodulatory molecules, including a structurally distinct equivalent of the mammalian transforming growth factor (TGF)-β cytokine, termed *H. polygyrus* TGF-β Mimic (Hp-TGM) [49,55]. Studies with TGF-β demonstrate that signaling induced by this molecule increased growth arrest and p53 and p21 expression [56], which leads us to hypothesize that the TGF-β mimic present in *H. polygyrus*-derived antigens, may drive the changes in cancer cell behavior that we report here through a common signaling pathway. Interestingly, although SEA was previously shown to inhibit CD4^+^ T cell proliferation and amplify TGF-β signaling [57], we found that the proliferation of CT26.WT cells was not affected by treatment with this antigen. This disparity may result due to the mechanism of action of the differing antigens; HES and Hp-TGM were reported to directly bind to TGF-β receptors and phosphorylate Smad, whereas SEA induced TGF-β expression and increased surface-bound TGF-β on CD4^+^ T cells [49,55,57]. Intriguingly, our results demonstrate that migration of human HCT116 cells decreased following exposure to *H. polygyrus*-derived antigens, which contrasted with increased migration seen in antigen-exposed murine CT26.WT cells. Analysis of human HCT116 CRC cells demonstrated that they have a mutated type II TGF-β receptor gene and cannot respond to TGF-β in order to undergo EMT, invasion, or migration [58]. A subsequent study demonstrated that HCT116 phosphorylated Smad in response to TGF-β but that this did not alter proliferation in these cells [59]. It is therefore conceivable that HCT116 cells are less responsive to the migration promoting effects of *H. polygyrus*-derived antigens and the TGF-β mimic contained within HES.

Our data provides the first evidence that antigen derived from *H. polygyrus* can alter human and murine CRC cell behavior. Further work would be required to test our hypothesis that Hp-TGM orchestrates this effect. This data provides an important basis for determining how parasite antigens influence cancer cell development in vivo and may have important implications for the etiology of colorectal cancer within parasite-endemic regions.

## 4. Materials and Methods

### 4.1. Heligmosomoides Polygyrus Antigen and HES Preparation

*H. polygyrus* somatic antigen (referred to as *H. polygyrus* antigen) and *H. polygyrus* excretory/secretory products (HES) were prepared using established methods described by Hewitson et al. and Johnston et al. [43,60]. In brief, male, 8-week-old, C57BL/6 mice were infected with 400 L3 *H. polygyrus* larvae by gavage, and adult worms were recovered 14 days post-infection. Adult worms were washed extensively before incubation in RPMI 1640 medium, supplemented with 2 mM L-glutamine, 100 U/mL penicillin, 100 U/mL streptomycin, and 100 µg/mL gentamicin. Pooled culture supernatants were filtrated into phosphate buffered saline PBS over a 3000 MW Amicon membrane (Merck Millipore, Darmstadt, Germany). The resulting HES was a well characterized preparation containing 374 proteins, novel O-linked glycoproteins, and small RNAs [43,45,61]. *H. polygyrus* antigen was prepared by uniformly homogenizing adult worms in PBS on ice using a Polytron homogenizer (Kinematica AG, Lucerne, Switzerland; 3 × 30 s pulses at speed 3, with 30 s intervals). The resulting mixture of 446 proteins was centrifuged at 13,000 *g* for 30 min, from which the supernatant was collected [43]. The protein concentration of HES and *H. polygyrus antigen* was quantified using a NanoDrop 1000 Spectrophotometer (Thermo Fisher Scientific, Wilmington, USA) before storing at −80 °C.

### 4.2. Cell Culture

The colorectal cancer (CRC) cell line CT26.WT (a murine N-nitroso-N-methylurethane (NNMU)-induced, undifferentiated fibroblast colon carcinoma cell line) and HCT116 (a human epithelial colon carcinoma cell line) were purchased from the American Type Culture Collection (ATCC). CT26.WT and HCT116 were maintained in complete RPMI 1640 and McCoy’s 5A modified medium, respectively, supplemented with 10% heat-inactivated fetal bovine serum (FBS), 100 U/mL penicillin, and 100 U/mL streptomycin. Cells were maintained at 37 °C in an atmosphere of 5% CO_2_ and 95% humidity.

### 4.3. Growth Curve Assay

The short-term growth of cells was monitored over a 3-day period, as previously described [62]**,** and 5 × 10^4^ cells were seeded per well, in triplicate, in a 24-well plate. Additionally, HES (10 µg), *H. polygyrus* antigen (10 µg), or 1× PBS was added to each of the required wells at the time of seeding. Live cells were grown in complete culture media and counted 24-, 48- and 72-h post-treatment using trypan blue exclusion.

### 4.4. 3-(4,5-dimethylthiazol-2-yl)-2,5-Diphenyltetrazolium Bromide (MTT) Assay

CRC mitochondrial activity was determined using MTT assays (Roche, Mannheim, Germany), and 8 × 10^3^ cells were seeded per well, in quadruplicate, in a 96-well plate. Additionally, HES (10 µg), *H. polygyrus* antigen (10 µg), or 1× PBS was added to each of the required wells for 24 h. A total of 0.5 mg/mL of MTT reagent was added to each well for 4 h, followed by the addition of 100 µL of solubilization solution. After an overnight incubation, the absorbance reading of each well was read at a wavelength of 595 nm. The absorbance of medium-only wells was subtracted from the absorbance of sample wells to obtain a final absorbance reading.

### 4.5. Bromodeoxyuridine (BrdU) Incorporation Assay

A BrdU incorporation assay was used in order to determine the effect of HES and *H. polygyrus* antigen on CT26.WT DNA synthesis and cell division, and 2 × 10^5^ cells were seeded on a sterile glass coverslip in a 35 mm dish, treated with HES (10 µg), *H. polygyrus* antigen (10 µg), or 1X PBS, and allowed to adhere overnight. A total of 10 µm BrdU (Sigma-Aldrich, Saint Louis, USA) was added to the medium for 8 h, followed by fixation with Carnoy’s fixative for 20 min at −20 °C. Cells were incubated in 2 M HCl for 1 h at 37 °C, neutralized in 0.1 M pH 8.5 borate buffer, and washed with PBS/0.5% Tween20. Cells were then incubated for 30 min at 37 °C in blocking buffer (5% sheep serum in PBS/0.5% Tween20). BrdU was detected with a mouse monoclonal anti-BrdU (clone: BMC9318, Roche, Mannheim, Germany) for 30 min at 37 °C, followed by a secondary IgG coupled to Alexa488 (RRID: AB_2534069, Thermo Fisher Scientific, Wilmington, USA) for 30 min at 37 °C. Cells were washed with PBS/0.5% Tween20, incubated in 1 µg 4’6-diamidino-2-phenylindole (DAPI) (Molecular Probes, Inc, Eugene, USA) for 10 min at room temperature, in the dark, and washed with PBS/0.5% Tween20. The coverslips were then mounted onto microscope slides, stored in the dark in a humidifying chamber overnight at 4 °C, and visualized by fluorescence microscopy using an Axio Vert.A1 Fluorescent microscope (Zeiss, Jena, Germany).

### 4.6. Schistosoma Mansoni Egg Antigen (SEA) Preparation

SEA was kindly gifted by Associate Professor Benjamin Dewals (University of Liège). Briefly, the livers of Naval Medical Research Institute (NMRI) mice infected with 200 cercariae were cut into pieces and individually incubated overnight in 20 mL of PBS 100 μg/mL collagenase IV. In order to collect the egg pellet, the homogenates were filtered and the suspension on top of the final 45 μm strainer was collected and centrifuged at 1400 rpm for 5 min at 20 °C. This was then centrifuged through a 20% Percoll solution, washed in PBS 1 mM EGTA/1 mM EDTA, and centrifuged through a 25% Percoll solution, and a further wash in PBS 1 mM EGTA/1mM EDTA was performed. The purified eggs were resuspended in PBS at a concentration of 100,000 eggs/mL before homogenizing on ice. The crude mixture was centrifuged at 2000× *g* for 20 min at 4 °C and the supernatant was then ultracentrifuged at 100,000× *g* for 90 min and sterilized through a 0.2 μm filter, before protein concentration determined by bicinchoninic acid (BCA) assay. The final preparation was stored at −70 °C.

### 4.7. Transwell Migration Assay

An established transwell migration assay, utilizing 24-well hanging inserts, fitted with an 8 µm pore size membrane (Merck Millipore, Darmstadt, Germany), was used to investigate CRC cell migration [62]. Additionally, 1 × 10^5^ serum starved cells were seeded in triplicate in media containing 1% FBS, onto the apical surface of each hanging insert, and placed into wells containing 10% FBS. Following treatment with HES (10 µg), *H. polygyrus* antigen (10 µg), or 1× PBS, the plates were incubated for 24 h. Post-incubation, the lower surface of the insert was fixed with methanol and stained with crystal violet. Excess crystal violet was washed off the insert using distilled water before release of the crystal violet stain into 50% acetic acid. The absorbance of this solution was then quantified at 595 nm using a Rayto RT-2100C Microplate Reader (Rayto, Shenzhen, China).

### 4.8. Western blotting

Western blotting was performed as previously described [63]. Primary antibodies used were as follows: mouse monoclonal anti-p21 (clone F-5, Santa Cruz Biotechnology, Inc), mouse monoclonal anti-p53 (clone DO-1, Santa Cruz Biotechnology, Inc, Texas, USA), mouse monoclonal β-catenin (clone 15B8, ThermoFisher Scientific, Wilmington, USA), and rabbit polyclonal anti-p38 MAP Kinase (Sigma-Aldrich, Saint Louis, USA). Signal was detected using peroxidase-conjugated goat anti-mouse or anti-rabbit antibodies (1:5000) and visualized by enhanced chemiluminescence (ECL) (Thermo Scientific, Carlsbad, USA or Advansta, R-03031-D25, San Jose, USA). Densitometry readings were obtained using ImageJ in order to determine changes in protein expression normalized to p38 (loading control) and expressed as a fold-change relative to control wells.

### 4.9. Ethics

Dispensation to perform research at the Institute of Infectious Disease and Molecular Medicine, Faculty of Health Sciences, University of Cape Town, was granted on 19 October 2015 by the South African Department of Agriculture, Forestry, and Fisheries, in terms of Section 20 of the Animal diseases act, 1984 (Act no. 35 of 1984): reference 12/11/17 under the title of research: “Worm power: Can helminths modify the development of colorectal cancer? AEC 015/001”. Approval for all animal procedures was given on 18 March 2015 by the Faculty of Health Science Animal Ethics Committee (Project 015/001) and was performed by researchers accredited by the South African Veterinary Council (AR15/13922).

### 4.10. Statistics

Data were assessed for normality and equal variance using the GraphPad Prism software (La Jolla, CA, USA). For comparison between the three treatment groups, a parametric one-way analysis of variance with Tukey’s multiple comparison or a nonparametric Kruskal–Wallis with Dunn’s multiple comparison was used. * *p* < 0.05, ** *p* < 0.01, and *** *p* < 0.001.

## Supplementary Materials

Supplementary Materials can be found at https://www.mdpi.com/1422-0067/21/21/7845/s1. Figure S1: *H. polygyrus*-derived antigens increase the expression of colorectal cancer (CRC) cell-cycle arrest proteins p53 and p21.

## Abbreviations

APC	Adenomatous Polyposis Coli
ATCC	American Type Culture Collection
BCA	Bicinchoninic Acid
BrdU	Bromodeoxyuridine
CAC	Colitis Associated Colorectal Cancer
CCA	Cholangiocarcinoma
CRC	Colorectal Cancer
DALY	Disability-Adjusted Life Years
DAPI	4’6-diamidino-2-phenylindole
ECL	Enhanced Chemiluminescence
EMT	Epithelial-Mesenchymal Transition
FBS	Foetal Bovine Serum
HES	*H. polygyrus* Excretory/Secretory Products
Hp-TGM	*H. polygyrus* TGF-β Mimic
IBD	Inflammatory Bowel Disease
LMIC	Low-and Middle-Income Countries
MTT	3-(4,5-dimethylthiazol-2-yl)-2,5-diphenyltetrazolium bromide
NMRI	Naval Medical Research Institute
PBS	Phosphate Buffered Saline
SD	Standard Deviation
SEA	*Schistosoma mansoni* egg antigen
STH	Soil-transmitted helminth
