# Supplementary material for: Gastrointestinal Nematode-Derived Antigens Alter Colorectal Cancer Cell Proliferation and Migration through Regulation of Cell Cycle and Epithelial-Mesenchymal Transition Proteins
Source: Int J Mol Sci. 2020 Oct 22;21(21):7845. doi: 10.3390/ijms21217845 (PMC7660063; doi:10.3390/ijms21217845)
Supplement: Supplementary file 1 [file ijms-21-07845-s001.pdf]

International Journal of
*Molecular Sciences*

MDPI

*Supplementary Materials*

# Gastrointestinal Nematode-Derived Antigens Alter Colorectal Cancer Cell Proliferation and Migration through Regulation of Cell Cycle and Epithelial-Mesenchymal Transition Proteins

**Brittany-Amber Jacobs [1], Sharon Prince [2] and Katherine Ann Smith [1,3,*]**

[1] Institute of Infectious Disease and Molecular Medicine, University of Cape Town, Cape Town 7925, South Africa; brittany-amber.jacobs@ludwig.ox.ac.uk
[2] Department of Human Biology, University of Cape Town, Cape Town 7925, South Africa; sharon.prince@uct.ac.za
[3] School of Medicine, Cardiff University, Cardiff CF14 3XN, UK; smithk28@cardiff.ac.uk
* Correspondence: smithk28@cardiff.ac.uk; Tel.: +44-2920-874-303

Received: 03 September 2020; Accepted: 19 October 2020; Published: date

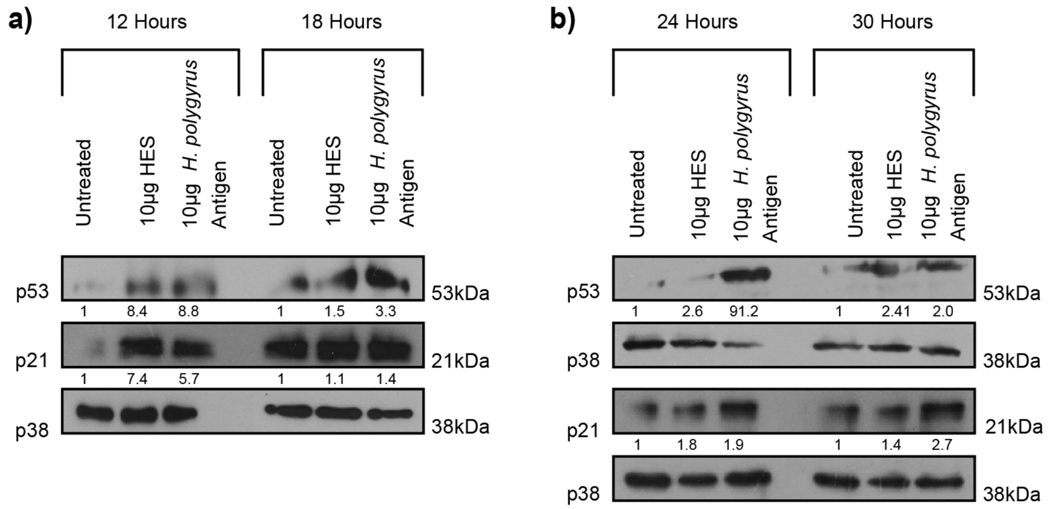


**Supplementary Figure 1**: *H. polygyrus*-derived antigens increase the expression of colorectal cancer (CRC) cell-cycle arrest proteins p53 and p21. Western blot analyses of p53, p21 and p38 (loading control) were performed following exposure of (a) CT26.WT cells for 12- and 18-hours and (b) HCT116 cells for 24- and 30-hours to 10 µg *H. polygyrus* antigen and 10 µg HES. Densitometry readings were obtained using ImageJ in order to determine the changes in protein expression of p53 and p21 normalised to p38 and expressed as a fold-change relative to control wells. This figure represents the second independent repeat of figure 3.
